# *Aedes nigrinus* (Eckstein, 1918) (Diptera, Culicidae), a new country record for England, contrasted with *Aedes sticticus* (Meigen, 1838)

**DOI:** 10.3897/zookeys.671.12447

**Published:** 2017-04-27

**Authors:** Ralph E. Harbach, Thom Dallimore, Andrew G. Briscoe, C. Lorna Culverwell, Alexander G.C. Vaux, Jolyon M. Medlock

**Affiliations:** 1 Department of Life Sciences, Natural History Museum, London, SW7 5BD, UK; 2 Biology Department, Edge Hill University, Ormskirk, Lancashire, L39 4QP, UK; 3 Department of Virology, Haartman Institute, University of Helsinki, Helsinki, Finland; 4 Medical Entomology & Zoonoses Ecology group, Emergency Response Department, Public Health England, Porton Down, Salisbury, SP4 0JG, UK

**Keywords:** Adults, bionomics, country records, diagnosis, identification, larvae, male genitalia, pupae

## Abstract

We report the discovery of *Aedes nigrinus* (Eckstein, 1918) in the New Forest of southern England, bringing to 36 the number of mosquito species recorded in Britain. Because it seems that this species has been misidentified previously in Britain as the morphologically similar *Aedes sticticus* (Meigen, 1838), the two species are contrasted and distinguished based on distinctive differences exhibited in the adult and larval stages. The pupa of *Ae. nigrinus* is unknown, but the pupa of *Ae. sticticus* is distinguished from the pupae of other species of *Aedes* by modification of the most recent key to British mosquitoes. The history of the mosquito fauna recorded in the UK is summarized and bionomical information is provided for the two species.

## Introduction

The number of mosquito species reported to occur in the United Kingdom has increased significantly since Stephens (1825) recognized the presence of 18 species, 14 of which were incorrectly identified or are currently recognized as synonyms of other species. Nearly a century later, Lang (1920) recorded 20 species, including four that were denoted with names that are currently synonyms of those species. Stephens (1825) recognized only two genera, *Culex* Linnaeus, 1758 for culicine species and *Anopheles* Meigen, 1818, whereas Lang (1920) used a number of generic names that are currently considered to be subgeneric names or synonyms of contemporary genera, including Culicella Felt, 1904 (subgenus of Culiseta Felt, 1904), Finlaya Theobald, 1903 (subgenus of Aedes Meigen, 1818), Ochlerotatus Lynch Arribálzaga, 1891 (subgenus of Aedes), *Taeniorhynchus* Lynch Arribálzaga, 1891 (synonym of subgenus Ochlerotatus of *Aedes*) and *Theobaldia* Neveu-Lemaire, 1902 (synonym of *Culiseta*). Following the generic classification of Edwards (1932), except for retaining *Taeniorhynchus*, which Edwards listed as a synonym of *Mansonia* Blanchard, 1901, Marshall (1938) described 29 British species belonging to six genera – *Aedes*, *Anopheles*, *Culex*, *Orthopodomyia* Theobald, 1904, *Taeniorhynchus* and *Theobaldia*. *Theobaldia* Neveu-Lemaire, 1902, being preoccupied by *Theobaldia* Fischer, 1885, was replaced by *Culiseta* Felt, 1904 (see Stone et al. 1959), which was in use by most American authors while *Theobaldia* was being used by European authors. In accordance with the classification of the Culicidae compiled in *A Catalog of the Mosquitoes of the World* (Knight and Stone 1977), Cranston et al. (1987) listed the occurrence of 33 species in Britain, five species of subfamily Anophelinae Grassi, 1900, all in genus *Anopheles*, and 28 species of subfamily Culicinae Meigen, 1818 belonging to five genera, *Aedes* (14 species), *Coquillettidia* Dyar, 1905 (1 species), *Culex* (5 species), *Culiseta* (7 species) and *Orthopodomyia* (1 species). Snow (1990) reduced the list to 32 species by appropriately recognizing *Cx. molestus* Forskål, 1775 as a “form” of *Cx. pipiens*. The list grew to 33 species with the addition of *Anopheles daciae* Linton, Nicolescu & Harbach, 2004 of the Maculipennis Complex based on molecular evidence (Linton et al. 2005), to 34 species with the discovery of *Aedes geminus* Peus, 1970 among museum specimens collected by J.F. Marshall and J. Staley around the Hayling Island area (Medlock and Vaux 2009) and then to 35 species with the recent detection of the invasive *Aedes albopictus* (Skuse, 1895) in southern England (Medlock et al. 2017). In the present paper, we report the discovery of *Aedes nigrinus* (Eckstein, 1918) in the New Forest of southern England, bringing to 36 the number of mosquito species recorded in Britain. Evidence indicates that this species has been misidentified in the past as the morphologically similar *Aedes sticticus* (Meigen, 1838); hence, the two species are contrasted herein. The history of the mosquito fauna recorded in the UK is summarized in Table 1, which includes, for completeness, the nominal species catalogued by Verrall (1901).

**Table 1.** Summary of the history of the mosquito fauna recorded in the UK.

Stephens (1825)	Verrall (1901)	Lang (1920) (20 species)	Marshall (1938) (29 species)	Cranston et al. (1987); Snow (1990) (32 species)	Today (36 species)
	*Aedes cinereus*; *Culex nigritulus*	*Aedes cinereus*	*Aedes cinereus*	Aedes (Aedes) cinereus	Aedes (Aedes) cinereus
					Aedes (Aedes) geminus **^1^**
	*Culex vexans*	*Ochlerotatus vexans*	*Aedes vexans*	Aedes (Aedimorphus) vexans	Aedes (Aedimorphus) vexans
*Culex cantans*; *Cx. maculatus*	*Culex annulipes*	*Ochlerotatus annulipes*	*Aedes annulipes*	Aedes (Ochlerotatus) annulipes	Aedes (Ochlerotatus) annulipes
		*Ochlerotatus waterhousei*	*Aedes cantans*	Aedes (Ochlerotatus) cantans	Aedes (Ochlerotatus) cantans
		*Ochlerotatus caspius*	*Aedes caspius*	Aedes (Ochlerotatus) caspius	Aedes (Ochlerotatus) caspius
*Culex domesticus* (in part); *Cx. nemorosus*	*Culex nemorosus*	*Ochlerotatus nemorosus*	*Aedes communis*	Aedes (Ochlerotatus) communis	Aedes (Ochlerotatus) communis
		*Ochlerotatus detritus*	*Aedes detritus*	Aedes (Ochlerotatus) detritus	Aedes (Ochlerotatus) detritus
	*Culex dorsalis*	*Ochlerotatus curriei*	*Aedes dorsalis*	Aedes (Ochlerotatus) dorsalis	Aedes (Ochlerotatus) dorsalis
	*Culex lutescens*		*Aedes flavescens*	Aedes (Ochlerotatus) flavescens	Aedes (Ochlerotatus) flavescens
			*Aedes leucomelas*	Aedes (Ochlerotatus) leucomelas	Aedes (Ochlerotatus) leucomelas
			*Aedes sticticus* **^2^**		Aedes (Ochlerotatus) nigrinus **^3^**
			*Aedes punctor*	Aedes (Ochlerotatus) punctor	Aedes (Ochlerotatus) punctor
	*Culex nigripes* (syn. var. sylvae)		*Aedes sticticus* **^2^**	Aedes (Ochlerotatus) sticticus	Aedes (Ochlerotatus) sticticus
*Culex ornatus* (in part)	*Culex diversus*; *Cx. rusticus*	*Ochlerotatus rusticus*	*Aedes rusticus*	Aedes (Ochlerotatus) rusticus	Aedes (Rusticoidus) rusticus
*Culex ornatus* (in part)	*Culex lateralis*; *Cx. ornatus*	*Finlaya geniculata*	*Aedes geniculatus*	Aedes (Finlaya) geniculatus	Aedes (Dahliana) geniculatus
					Aedes (Stegomyia) albopictus ^4^
			*Anopheles algeriensis*	Anopheles (Anopheles) algeriensis	Anopheles (Anopheles) algeriensis
*Anopheles maculipennis* *s.l.***^5^**	*Anopheles maculipennis s.l.^5^*	*Anopheles maculipennis* *s.l.***^5^**	*Anopheles maculipennis* *s.l.***^5^**	Anopheles (Anopheles) atroparvus	Anopheles (Anopheles) atroparvus
	*Anopheles bifurcatus*	*Anopheles bifurcatus*	*Anopheles claviger*	Anopheles (Anopheles) claviger	Anopheles (Anopheles) claviger
					Anopheles (Anopheles) daciae **^6^**
				Anopheles (Anopheles) messeae	Anopheles (Anopheles) messeae
	*Anopheles nigripes*	*Anopheles plumbeus*	*Anopheles plumbeus*	Anopheles (Anopheles) plumbeus	Anopheles (Anopheles) plumbeus
	*Taeniorhynchus richiardii*	*Taeniorhynchus richiardii*	*Taeniorhynchus richiardii*	Coquillettidia (Coquillettidia) richiardii	Coquillettidia (Coquillettidia) richiardii
				Culex (Barraudius) modestus	Culex (Barraudius) modestus
			*Culex molestus*	Culex (Culex) pipiens molestus **^7^**	
*Anopheles bifurcatus*; *Cx. bicolor*; *Cx. domesticus* (in part); *Cx. lutescens*; *Cx. marginalis*; *Cx. pipiens*; *Cx. punctatus*; *Cx. rufus*; *Cx. sylvaticus*	*Culex pipiens* (syn. *Cx. ciliaris*)	*Culex pipiens*	*Culex pipiens*	Culex (Culex) pipiens	Culex (Culex) pipiens
				Culex (Culex) torrentium	Culex (Culex) torrentium
		*Culex apicalis*	*Culex apicalis*	Culex (Neoculex) territans	Culex (Neoculex) territans
				Culiseta (Allotheobaldia) longiareolata	Culiseta (Allotheobaldia) longiareolata
*Culex fumipennis*	*Culex cantans* (syn. *Cx. fumipennis*)	*Culicella fumipennis*	*Theobaldia fumipennis*	Culiseta (Culicella) fumipennis	Culiseta (Culicella) fumipennis
			*Theobaldia litorea*	Culiseta (Culicella) litorea	Culiseta (Culicella) litorea
	*Culex morsitans*	*Culicella morsitans*	*Theobaldia morsitans*	Culiseta (Culicella) morsitans	Culiseta (Culicella) morsitans
			*Theobaldia alaskaensis*	Culiseta (Culiseta) alaskaensis	Culiseta (Culiseta) alaskaensis
*Culex affinis*; *Cx. annulatus*; *Cx. calopus*	*Culex annulatus*	*Theobaldia annulata*	*Theobaldia annulata*	Culiseta (Culiseta) annulata	Culiseta (Culiseta) annulata
			*Theobaldia subochrea*	Culiseta (Culiseta) subochrea	Culiseta (Culiseta) subochrea
	*Culex pulchripalpis* [*sic*]	*Orthopodomyia albionensis*	*Orthopodomyia pulcripalpis*	*Orthopodomyia pulcripalpis*	*Orthopodomyia pulcripalpis*

**^1^** Recorded by Medlock and Vaux (2009).

**^2^** At least in part, see Discussion.

**^3^** Recorded herein.

**^4^** Recorded by Medlock et al. (2017).

**^5^** Also includes *Anopheles daciae* and *An. messeae*.

**^6^** Recorded by Linton et al. (2005).

**^7^** Synonym of *Culex pipiens*; recognized as a “form” of *Cx. pipiens* by Snow (1990).

## Materials and methods

### Mosquitoes

Mosquitoes were collected as larvae and individually reared to adults. Larvae of adults identified as *Aedes nigrinus* were collected on 22 May 2016 at Beaulieu Airfield (50°48.53'N; 1°29.79'W and 50°48.11'N; 1°30.83'W), New Forest, Hampshire, England. Larvae of *Ae. sticticus* were collected on 10 May 2011 in Hurcott Wood (32°23.92'N; 2°12.73'W), Kidderminster, Worcestershire, England. The larval and pupal exuviae of *Ae. nigrinus* were lost; those of *Ae. sticticus* were mounted in Euparal on microscope slides. Adults were mounted on points on insect pins. Dissected male genitalia of both species were cleared in 5% NaOH for 2 h at 50°C and slide-mounted in Euparal. The pinned adults were examined under simulated natural light with an Olympus SZ6045 stereomicroscope. The dissected genitalia were studied with an Olympus BX50 compound microscope fitted with differential interference contrast optics. Digital images of wings and genitalia were taken with a Canon 550D digital camera mounted on a Leica M125 stereomicroscope and a Zeiss Axioskop compound microscope, respectively; Helicon Focus version 3.03 software (Helicon Soft Ltd, Kharkov, Ukraine) was used to obtain extended-focus images. The anatomical terminology of Harbach and Knight (1980, 1982), revised and updated in the Anatomical Glossary of the Mosquito Taxonomic Inventory (http://mosquito-taxonomic-inventory.info/node/11027), is used in the descriptions and illustrations.

Abbreviations for morphological structures indicated in figures:

**BDL** basal dorsomesal lobe

**C** costa

**R_1_** radius-one

**Re** remigium

**Sc** subcosta

**1A** anal vein

### DNA extraction, amplification and sequencing

DNA was extracted from two legs from each of five adults of *Ae. nigrinus* using the DNeasy Blood & Tissue Kit (Qiagen, Hilden, Germany) in accordance to the manufacturer’s instructions. Amplification of the mitochondrial cytochrome oxidase subunit I (*COI*) gene and the nuclear internal transcriber spacer 2 (ITS2) region of ribosomal DNA was carried out using the following primers: 5’- GGATTTGGAAATTGATTAGTTCCTT-3’ (COIF) and 5’- AAAAATTTTAATTCCAGTTGGAACAGC-3’ (COIR) (Chan et al. 2014), 5’- TGTGAACTGCAGGACACATG-3’ (ITS2F) and 5’- ATGCTTAAATTTAGGGGGTA-3’ (ITS2R) (Walton et al. 2007). PCR was undertaken using Phusion High-Fidelity PCR Master Mix (New England Biolabs, Ipswich, MA, USA) and products were purified using Isolate II PCR and Gel spin columns (Bioline Reagents Limited, London, UK). Subsequent sequencing reactions were undertaken using the BigDye™ Terminator v. 3.1 Cycle Sequencing Kit (Applied Biosystems Inc., Foster City, CA, USA) and sequenced on a 3500 Series Genetic Analyzer. Analysis of the sequence data was carried out using MEGA7 (Kumar et al. 2016). The exact location and size of the ITS2 region was determined by annotation of the 5.8S/28S flanking regions using the ITS2 database (http://its2.bioapps.biozentrum.uni-wuerzburg.de/).

## Results

Adults reared from larvae collected in the New Forest were initially questionably identified as specimens of *Ae. sticticus* using the keys to British mosquitoes provided by Cranston et al. (1987), consequently we ran adults and male genitalia through the keys to European species of *Aedes* and *Ochlerotatus* included in Becker et al. (2010). Based on differential characters of the antennae, wings and abdominal terga of females and characteristics of the apical and basal dorsomesal lobes of the gonocoxites of males, the specimens keyed to *Ae. nigrinus*. This engendered a comparison with specimens collected in Hurcott Wood five years earlier that were identified as *Ae. sticticus* using the keys to adults and larvae contained in Cranston et al. (1987) and Becker et al. (2010). Based on this comparison, there was little doubt that the New Forest specimens were correctly identified as *Ae. nigrinus*. Incidentally, while examining older British mosquito publications, we noted that Marshall (1938) had examined specimens from the New Forest and the male genitalia he illustrated for *Ae. sticticus* appear to be those of *Ae. nigrinus*.

To confirm the morphological identification of *Ae. nigrinus*, we sequenced part of the mitochondrial cytochrome oxidase subunit I gene and the internal transcribed spacer 2 region of ribosomal DNA from specimens collected in the New Forest and Hurcott Wood. Sequences generated in this study were interrogated against the NCBI non-redundant nucleotide database via the BLAST algorithm (Altschul et al. 1990). No ITS2 sequences for *Ae. nigrinus* were available for comparison, but our sequence shared a 95% sequence identity (289/305 bases) to *Ae. sticticus* (KF535079). Following alignment, this difference was accounted for by a number of consistent SNP sites and a six-nucleotide insertion/deletion 207 bases into our sequence. The *COI* sequences of New Forest specimens also returned high BLAST scores for *Ae. sticticus*, but only because the query coverage was higher due to the primer pairs used for amplification. The closest match recognized by identity score was *Ae. nigrinus* (98–99% compared with 96–97% for *Ae. sticticus*). These results support the morphological identification of *Ae. nigrinus* in the UK.

## Discussion

### Morphology and identification

**Figure 1.**
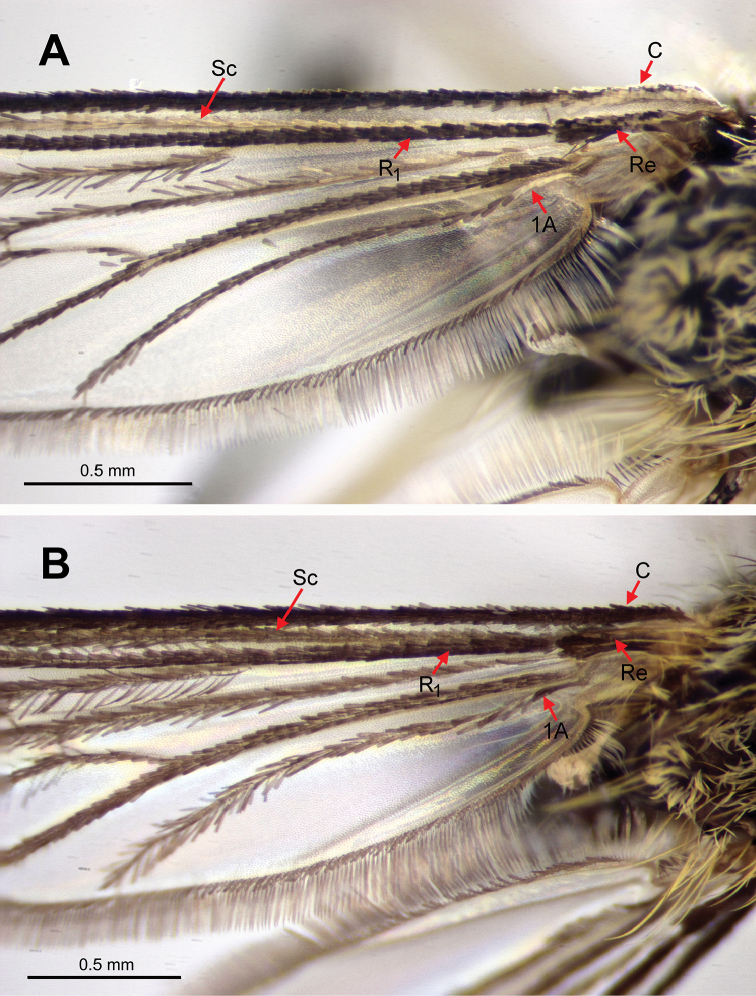
Proximal half of the right wing of female mosquitoes. **A** *Ae. nigrinus*, showing the presence of pale scaling on the costa, subcosta, remigium and anal vein **B** *Ae. sticticus*, showing the absence of pale scaling.

**Figure 2.**
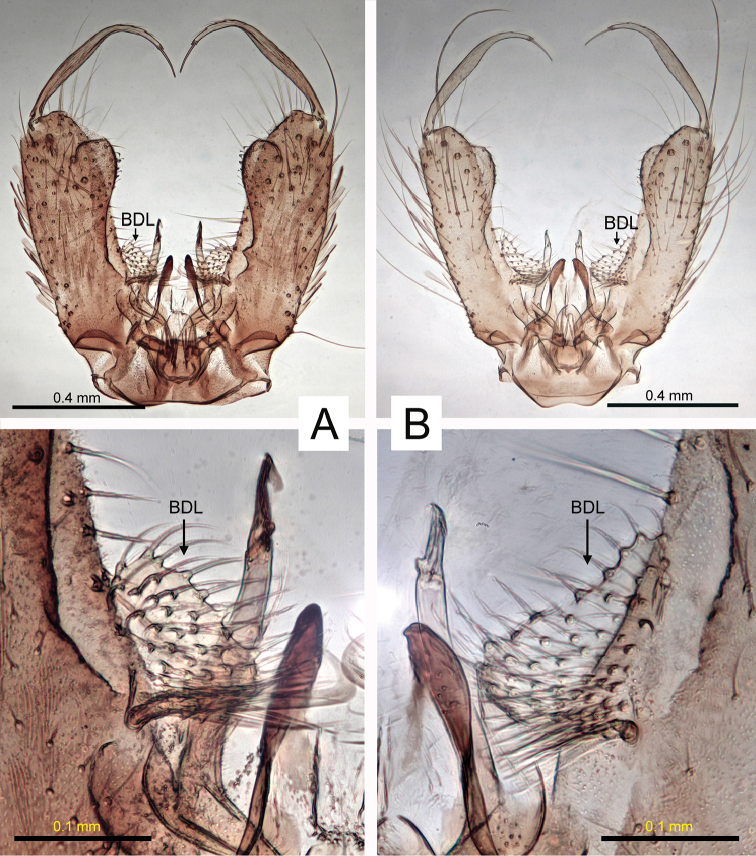
Doral aspects (pre-rotation sense) of the male genitalia of *Ae. nigrinus* (**A**) and *Ae. sticticus* (**B**), which are distinguished by the shape of the basal dorsomesal lobe (BDL).

It is unfortunate that the larval and pupal exuviae of mosquitoes reared from larvae collected in the New Forest were lost as these would have aided the identification of *Ae. nigrinus*. Further field work will be conducted to obtain the immature stages. However, a number of morphological differences are robust enough to distinguish the two species. To aid future identification, the wings and the male genitalia of *Ae. nigrinus* and *Ae. sticticus* are illustrated for comparison in Figures 1 and 2, respectively. The principal features that distinguish the two species are as follows.

*Aedes nigrinus*. A dark mosquito, integument and dark scaling black, well contrasted with pale scaling. Female: first antennal flagellomere and dorsal surface of pedicel black; wing with pale (white) scaling (Fig. 1A) at base of costa (C), length of subcosta (Sc), on remigium (Re) (not evident in figure), base of radius-one (R_1_) (not evident in figure) and base of anal vein (1A); abdominal terga with basal pale bands slightly constricted medially, sometimes reduced to lateral patches on tergum VII. Male genitalia (Fig. 2A): basal dorsomesal lobe of gonocoxite more or less globoid in dorsal (tergal) view. Fourth-instar larva (see Natvig 1948, Becker et al. 2010): setae 5,6-C usually single, rarely double; comb usually with 12–16 scales, rarely with more than 20.

*Aedes sticticus*. A slightly paler mosquito, integument and dark scaling dark brown to brownish black, less well contrasted with pale scaling. Female: first antennal flagellomere and dorsal surface of pedicel yellowish brown; wing entirely dark-scaled (Fig. 1B); abdominal terga mostly without complete basal pale bands, bands on terga II–IV narrow if present, more distal terga with triangular basolateral pale patches. Male genitalia (Fig. 2B): basal dorsomesal lobe more or less crescentic in dorsal (tergal) view, distomesal surface slightly concave. Fourth-instar larva (see Natvig 1948, Becker et al. 2010): setae 5,6-C usually with 2–4 branches, seta 6-C occasionally single; comb with 19–27 scales.

The female, male and fourth-instar larval stages of *Ae. nigrinus* and *Ae. sticticus* have been described, although not completely (Natvig 1948, Becker et al. 2003, 2010), but no attention has been given to the pupal stage of either species. The pupa of *Ae. nigrinus* remains unknown, but it is possible to distinguish the pupa of *Ae. sticticus* from other species included the key of Cranston et al. (1987) with the following modification of couplet 20 (measurements corrected and wording changed to reflect current usage of morphological terminology).

**Table:** 

20(18)	Paddle marginal spicules longer than 10 μm; seta 1-Pa single; paddle length usually greater than 0.85 mm; abdominal length greater than 3.5 mm	***Ae. punctor***
–	Paddle marginal spicules shorter than 10 μm; seta 1-Pa single or double; paddle length usually less than 0.85 mm; abdominal length less than 3.5 mm	**20a**
20a (20)	Seta 3-III branched; seta 1-Pa double	***Ae. dorsalis***
–	Seta 3-III single; seta 1-Pa single	***Ae. sticticus***

### Bionomics and distribution

*Aedes nigrinus* and *Ae. sticticus* were both originally described from localities in Germany (Knight and Stone 1977). As far as known, the distribution of *Ae. nigrinus* is limited to an area that extends from southern England (new record reported herein) and France to eastern Russia and from southern Scandinavia and Finland southward to Germany whereas *Ae. sticticus* is broadly distributed in northern areas of the Holarctic Region (Knight and Stone 1977, Becker et al. 2010). According to Cranston et al. (1987), *Ae. sticticus* has a “patchy distribution” in Europe and “is rare in Britain”. The few specimens present in the NHM collection lack basal pale bands with only lateral pale spots present. Such specimens, like most reared from larvae collected in Hurcott Wood, could be misidentified as *Ae. geniculatus* (Olivier, 1791) in the key of Cranston et al. (1987).

The occurrence of *Ae. sticticus* in Britain was first recorded by Marshall (1938), who listed the New Forest among other localities where the species had been collected. The New Forest record was later accepted without question by Cranston et al. (1987) and Snow (1990) and is the only record of the species in southern England (Snow et al. 1998). Since we now know that the New Forest record of Marshall refers to *Ae. nigrinus* (see above), the New Forest should not be listed as an occurrence record for *Ae. sticticus* until this species is definitely known to occur there.

The New Forest became a royal forest more than 950 years ago and is the largest remaining tract of unenclosed pasture land, heathland and forest in England. The land is dominated by gravel, sand and clay that was deposited during the Palaeogene Period of the Cenozoic Era (23.03–65.5 Mya). Many sites near the Beaulieu airfield where *Ae. nigrinus* was collected contain extensive areas of water-logged, marshy bogs and mires where the clay creates an impervious layer of saturated ground. The airfield was established during World War I, closed in 1919, re-opened again in 1942 and operated as an airfield for a further 15 years. Since 1959, the area has returned to an open heathland with open mireland habitat fringing the main airfield site.

*Aedes sticticus* is primarily associated with floodplains of rivers in forested areas (Cranston et al. 1987, Wood et al. 1979, Becker et al. 2010). In parts of Sweden, it contributes to a significant biting issue and is the subject of an extensive aerial mosquito control programme (Schäfer and Lundström 2009). In other parts of its range in central Europe, it also contributes to significant biting, along with *Aedes vexans* (Meigen, 1830), in floodwater areas along the Dyje River on the border of the Czech Republic and Austria, and is implicated in the transmission of Tahyna virus (Hubálek et al. 2010, Berec et al. 2014). Larvae of this species were found in a shallow floodwater pool in Hurcott Wood in early May and a few adults were collected in June in forest at Woodwalton Fen, Cambridgeshire. In contrast, *Ae. nigrinus* is mainly associated with floodwaters in more open terrain, such as meadows (Becker et al. 2010). Larvae of this species were found at the margins of small ponds in an open area of heath within the New Forest National Park in May, and a single adult male was collected in the same area in September.
